# Valorization of Enzymatic Hydrolysis Residues from Corncob into Lignin-Containing Cellulose Nanofibrils and Lignin Nanoparticles

**DOI:** 10.3389/fbioe.2021.677963

**Published:** 2021-04-16

**Authors:** Rui Xu, Haishun Du, Hui Wang, Meng Zhang, Meiyan Wu, Chao Liu, Guang Yu, Xinyu Zhang, Chuanling Si, Sun-Eun Choi, Bin Li

**Affiliations:** ^1^Tianjin Key Laboratory of Pulp and Paper, Tianjin University of Science and Technology, Tianjin, China; ^2^Key Laboratory of Biofuels, Qingdao Institute of Bioenergy and Bioprocess Technology, Chinese Academy of Sciences, Qingdao, China; ^3^Department of Chemical Engineering, Auburn University, Auburn, AL, United States; ^4^Department of Forest Biomaterials Engineering, College of Forest and Environmental Sciences, Kangwon National University, Chuncheon, South Korea

**Keywords:** lignin-containing cellulose nanofibrils, enzymatic hydrolysis residues, homogenization, corncob, lignin nanoparticles

## Abstract

As a kind of biomass waste, enzymatic hydrolysis residues (EHRs) are conventionally burned or just discarded, resulting in environmental pollution and low economic benefits. In this study, EHRs of corncob residues (CCR) were used to produce high lignin-containing cellulose nanofibrils (LCNFs) and lignin nanoparticles (LNPs) through a facile approach. The LCNFs and LNPs with controllable chemical compositions and properties were produced by tuning the enzymolysis time of CCR and the followed homogenization. The morphology, thermal stability, chemical and crystalline structure, and dispersibility of the resultant LCNFs and LNPs were further comprehensively investigated. This work not only promotes the production of lignocellulose-based nanomaterials but also provides a promising utilization pathway for EHRs.

## Introduction

In recent years, along with the increasing concerns derived from fossil resource dependence and environmental pollution, lignocellulosic biomass has been regarded as an alternative source of biofuels and bio-based products because of its large amount of stock and renewability (Xu et al., 2016; Li et al., 2019, 2020; Lin et al., 2020; Yang et al., 2020; Liu K. et al., 2021; Zheng et al., 2021b). It is worth noting that agricultural residues such as wheat straw, corn stalk, bagasse, and corncob are renewable lignocellulosic biomass yet not properly managed or utilized (Anwar et al., 2019). It has been reported that approximately 900 million tons of agricultural wastes were produced in China each year (Liu et al., 2019; Xu et al., 2019; Chen et al., 2020). However, most of the agricultural wastes are often disposed by burning, which results in low economic benefits and causes severe environmental pollution. Recently, burning of agricultural waste is strictly forbidden to protect the environment and achieve the target carbon neutrality in China. Thus, it is of great importance to valorize these agricultural wastes into valuable products by using sustainable and environmentally friendly processes (Xu et al., 2020).

Recent studies suggested that converting the cellulose and hemicellulose of biomass to monomeric sugars and biofuels could be a great road to meeting the urgent need for renewable alternative carbon resources (Robertson et al., 2017; Huang et al., 2019; Lin et al., 2019). Among the agricultural wastes, corncob has been widely concerned for its richness in hemicellulose, which is often used to produce xylose, xylitol, xylo-oligosaccharides, or furfural (Si et al., 2009b, 2013b; Zhang et al., 2014; Hu et al., 2016; Liu et al., 2016; Huang et al., 2020b). However, a large amount of corncob residue is produced and it is usually used for burning to generate heat or electricity, cultivation, feed, or just discarded. Yet, corncob residues contain abundant cellulose and lignin, which provide the possibility of creating useful materials from waste biomass, which would enable us to replace artificial and non-renewable resources with renewable resources (Kaneko et al., 2006; Si et al., 2009a, 2013a). Also, enzymatic hydrolysis of corncob residue is commonly used to produce sugar/ethanol (Xie et al., 2018b; Xu et al., 2021). However, the high production cost of enzymatic hydrolysis prevents its large-scale utilization (Lin and Lu, 2021; Liu H. Y. et al., 2021). Thus, apart from optimizing the enzymolysis process parameters, making better use of the solid residues produced after enzymatic hydrolysis could be a feasible method to reduce the total cost, based on the concept of integrated biorefinery.

As a kind of biomass waste, enzymatic hydrolysis residues (EHRs) are conventionally burned for heat and power generation, resulting in low economic benefits. Actually, the EHRs contain a significant amount of lignin, a small amount of cellulose, as well as some extractives and ash, and a trace amount of hemicellulose. As a primary starting material, the EHRs are qualified to produce lignocellulosic nanomaterials (Khan et al., 2018). Moreover, the abundant lignin existing in EHRs would play a good role in some properties such as thermal stability, antioxidation properties, hydrophobicity, and stabilization (Peng et al., 2018; An et al., 2019). By the way, in the actual industrial production, high solid enzymatic hydrolysis residue has more universal application. Thanks to the special structure of EHRs, the fiber is in a scattered and short form with a significant amount of lignin. Usually, fiber with a high content of lignin is difficult to disintegrate down to nanoscale due to the cross-linked barrier properties of lignin.

Lignocellulosic nanomaterials have received much attention at the forefront of bio-based economy in which renewable biomass is used as the raw material for the production of various consumer products (Salas et al., 2014; Song, 2019; Dai et al., 2020a). Recently, cellulose nanomaterials, mainly including cellulose nanocrystals (CNCs) and nanofibers (CNFs), have attracted rapidly growing interest from both academic and industrial researchers due to their superior physiochemical properties (Du et al., 2019; Huang et al., 2020a; Liu et al., 2020; Miao et al., 2020; Wang P. et al., 2020). However, the preparation of cellulose nanomaterials is facing several challenges such as complicated purification and pretreatment process, large amount of chemical and energy consumption, and environmental issues, which limited their large-scale applications (Xie et al., 2018a; Dai et al., 2020b; Wang H. et al., 2020). Fortunately, recent studies showed that lignin-containing cellulose nanomaterials can be obtained directly from raw materials via sustainable and low-cost approaches, and the presence of lignin endows the LCNM with many advantages such as improved thermal stability, UV-blocking performance, and water barrier property (Nair et al., 2017; Wang et al., 2018; Farooq et al., 2019; Hong et al., 2020). Especially in the last few years, lignin-containing cellulose nanofibrils (LCNFs) have been gradually arousing people’s interest. The preparation of LCNFs was confirmed by various approaches from different lignocellulose biomass including unbleached thermomechanical pulp, corn husk, and tobacco stalks, among others (Bian et al., 2017; Hu et al., 2018; Wang et al., 2018; Wen et al., 2019). In addition, lignin nanoparticles (LNPs) have gained significant interest among researchers in recent years, which will play a vital role in promoting lignin valorization (Chauhan, 2020; Dong et al., 2020a). It should be noted that EHRs contain much more lignin compared to unbleached pulp and most of lignocellulosic biomass (Huang et al., 2018), and it is expected that LCNFs with high lignin content or LNPs can be produced from EHRs.

In this work, the preparation of EHRs with different enzymatic hydrolysis times (48, 72, and 108 h, respectively, for comparison) was done in accordance with the previous work of our group (Chi et al., 2019), and subsequently, high-pressure homogenization treatment of the obtained EHR samples was carried out to produce LCNFs or LNPs. The physiochemical properties of the obtained LCNFs and LNPs were further comprehensively investigated. This work will promote the development of lignocellulose-based nanomaterials and provide a promising pathway for the full utilization of agricultural wastes.

## Materials and Methods

### Materials

Corncob residue (CCR) was purchased from Futaste Co., Ltd. (China), where corncob was used as raw material for the manufacture of xylose and xylitol using dilute acid hydrolysis. All the chemicals (e.g., KOH, H_2_SO_4_) used in this study were purchased from Sinopharm Chemical Reagent Co. Ltd. and used directly without further purification. Cellulase enzyme was obtained from Qingdao Vland Biotech Inc. The activity of cellulase was 85 FPU mL^–1^, which was measured by the standard procedure (Ghose, 1987) and the protein content of cellulase was 80 mg-protein per mL, as determined by the standard Bradford method.

### Preparation of LCNFs or LNPs

#### Alkaline Pretreatment of CCR

To improve the enzymatic hydrolysis efficiency of cellulose and effectively utilize the alkali lignin components, the as-received CCR was pretreated with 16 wt.% KOH at 70°C for 90 min following the procedure reported previously (Chi et al., 2019). After alkaline pretreatment, the collected solid was washed with distilled water to neutral pH and stored at 4°C.

#### Enzymatic Hydrolysis

Enzymatic hydrolysis of the alkaline-pretreated CCR was conducted with a high solid content (20%) under 50°C for different hydrolysis times (48, 72, and 108 h, respectively), and the enzyme dosage for saccharification was 15 FPU/g-cellulose. Finally, the EHRs were obtained by filtration and washed to neutral pH and stored at 4°C.

#### High-Pressure Homogenization

The obtained EHR samples were converted to LCNFs or LNPs using a high-pressure homogenizer (GYB49-10s, China). The EHRs treated in different enzymatic hydrolysis times (named EHRs-48 h, EHRs-72 h, and EHRs-108 h, respectively) were homogenized at a concentration of 2 wt.% in deionized water for three times at 30 MPa and then five times at 60 MPa. The obtained products were noted as LCNFs-48 h, LCNFs-72 h, and LNPs-108 h, respectively.

### Analytical Method for Composition

The composition of EHRs was determined following the National Renewable Energy Laboratory procedure (NREL). A dried EHR sample (0.30 g) was treated with 3 mL of 72% (w/w) H_2_SO_4_ at 30°C for 60 min. Then, the acid concentration was diluted to 4% (w/w) by adding deionized water, and the samples were further hydrolyzed at 121°C for 60 min. The residue (Klason lignin) was filtered with deionized water under vacuum and then dried at 105°C to constant weight. The carbohydrate content in the supernatant was quantified by high-performance liquid chromatography system (HPLC, Waters-1525, United States) analysis. The contents of cellulose and hemicellulose of EHR samples were calculated based on the amount of corresponding monomeric sugars (Sluiter et al., 2008).

### Morphology Analysis

The microstructure of samples was investigated by an S-4800 field-emission scanning electron microscope (SEM, Hitachi, Japan). After homogenization, the samples of LCNFs and LNPs were freeze dried. The freeze-dried samples were sprinkled on a conductive adhesive tape mounted on a specimen stub and coated with gold by a sputter coater (E-1045, Hitachi, Tokyo, Japan) before observation.

A transmission electron microscope (TEM, Hitachi, H-7650) was also used to investigate the microstructure of samples. LCNF or LNP suspensions with a concentration of 0.03% were deposited on carbon-coated TEM grids. After the drying process, the specimen was stained with 2% uranyl acetate solution for 1.5 h. Then, the excess staining solution was removed, and the samples were dried at ambient before characterization.

### Thermogravimetric (TG) Analysis

The thermal stability of the freeze-dried samples was evaluated using a TGA Q600 (TA Instruments, United States) instrument at temperatures ranging from 25 to 550°C. The experiment was carried out under a nitrogen atmosphere at a heating rate of 8°C/min (Yang et al., 2007).

### Fourier Transform Infrared Spectroscopy (FTIR)

FTIR spectra of EHR samples were determined using a FTIR spectrometer (Nicolet-6700, America) with a wavenumber region of 4,000–500 cm^–1^. Freeze-dried samples were diluted with KBr using a KBr tableting process before FTIR analysis.

### X-Ray Diffraction (XRD) Analysis

XRD analysis was conducted using an ADVANCE D8 X-ray diffractometer (Bruker Co., Germany) equipped with a Ni-filtered Cu Kα radiation operated at 40 kV and 30 mA. Briefly, 0.2 g freeze-dried samples was compressed into a pellet to record patterns from 5° to 60° of diffraction with a scan rate of 4°/min. The crystallinity index (CrI) was calculated according to Segal’s method (Segal et al., 1959) with the subtraction of background of glass.

### Zeta Potential and Dynamic Light Scattering (DLS)

The zeta potential and DLS analyses of LCNF and LNP suspensions were detected by a microscopic electrophoresis apparatus (Nano Brook 90Plus Zeta, America) and was calculated by the electrophoretic mobility. All the samples were ultrasonically treated for 20 min before analysis, and the solid concentration was around 0.05 wt.%. The measurements were conducted in triplicate for each sample, and the average data was reported.

## Results and Discussion

### Enzymatic Hydrolysis of CCR

**FIGURE 1:**
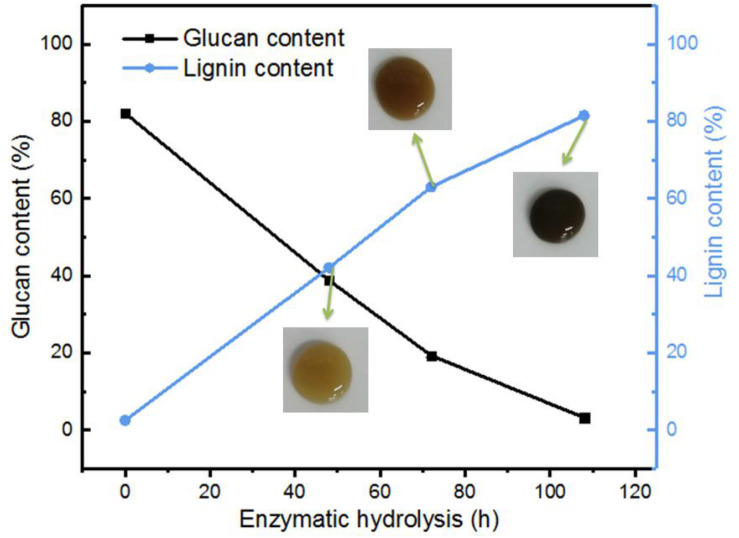
Lignin and glucan content in the EHRs after different enzymatic hydrolysis times.

As mentioned in the experiment part, the corncob after dilute acid hydrolysis and the followed alkaline treatment were used as the raw material in this study. As indicated in Table 1, the raw material (alkaline pretreated CCR) contains majority of cellulose with a small portion of residual xylan, lignin, and extractives, which could be a great feedstock for the preparation of glucose by enzymatic hydrolysis. Figure 1 shows the change of lignin and glucan contents in the EHRs with different enzymatic hydrolysis times. It can be clearly seen that with the increase of enzymolysis time, the content of glucan in the EHRs decreased dramatically, while the relative proportion of lignin significantly increased. The insert photos of the collected suspensions of the nanomaterial after homogenization vividly demonstrate the color change, which correlates with the lignin content. Table 1 gives a detailed description of the changes of each component in the EHRs. We can easily see that the cellulose and lignin account for most of the compositions of the collected EHRs for all the samples. Also, the content of xylan and extractives did not change greatly with enzymolysis time due to the strong specificity of the enzyme. Notably, the glucan content in the EHRs is only around 3.2% after enzymatic hydrolysis for 108 h. Depending on the chemical composition of the collected EHRs after enzymatic hydrolysis, we marked the samples prepared from EHRs-48 h and EHRs-72 h as LCNFs and the sample prepared from EHRs-108 as LNPs, respectively.

**TABLE 1:** Chemical composition of raw material and EHRs treated at different enzymatic hydrolysis times.

Samples	Extractives	Glucan	Xylan	Lignin
	content (%)	content (%)	content (%)	content (%)
Raw material	5.3 ± 1.1	82.2 ± 0.9	3.4 ± 0.2	2.5 ± 0.2
EHRs-48 h	8.7 ± 0.8	38.8 ± 0.6	2.6	42.1 ± 0.6
EHRs-72 h	7.1 ± 1.0	19.3 ± 0.5	2.4 ± 0.2	63 ± 0.7
EHRs-108 h	5.9 ± 0.6	3.2 ± 0.9	2.5 ± 0.1	81.5 ± 1

### Morphologies of LCNFs and LNPs

**FIGURE 2:**
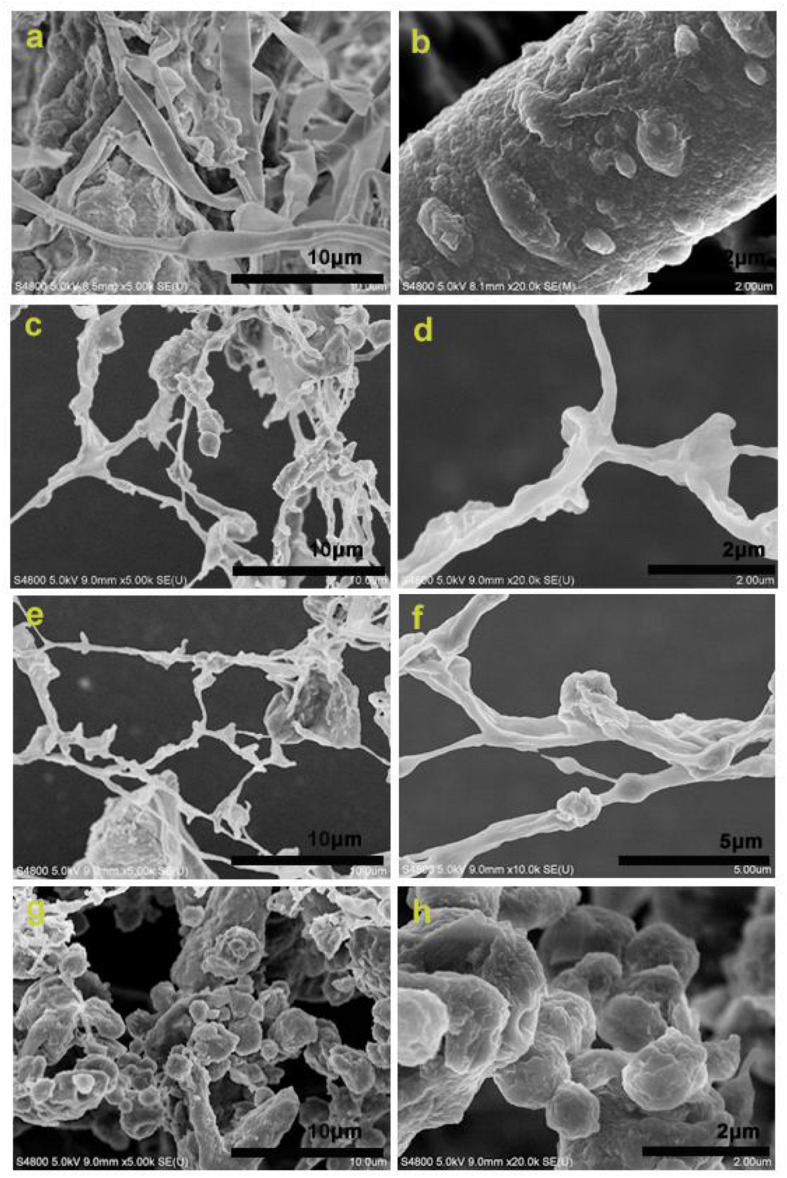
SEM images of the raw material **(a,b)**, LCNFss-48 h **(c,d)**, LCNFs-72 h **(e,f)**, and LNPs-108 h **(g,h)**.

Figures 2a,b show the morphology of the raw material (the alkaline-pretreated CCR) before enzymolysis. The cellulose fibers and lignin can be clearly seen, and there are some lignin particles anchored on the surface of cellulose fibers. From Figures 2c,d, it can be observed that the LCNFs-48 h was rich in nanofibers, and there were many irregular LNPs adhering on the nanofiber surface. Figures 2e,f display the morphology of LCNFs-72 h. According to these two images, the existence of nanofibers and LNPs on the surface of nanofibers could be seen clearly. Figures 2g,h show the morphology of LNPs-108 h, only a small number of nanofibers can be seen, and the LNPs show an obvious aggregation state (it is speculated to be the aggregate of LNPs before freeze-drying).

**FIGURE 3:**
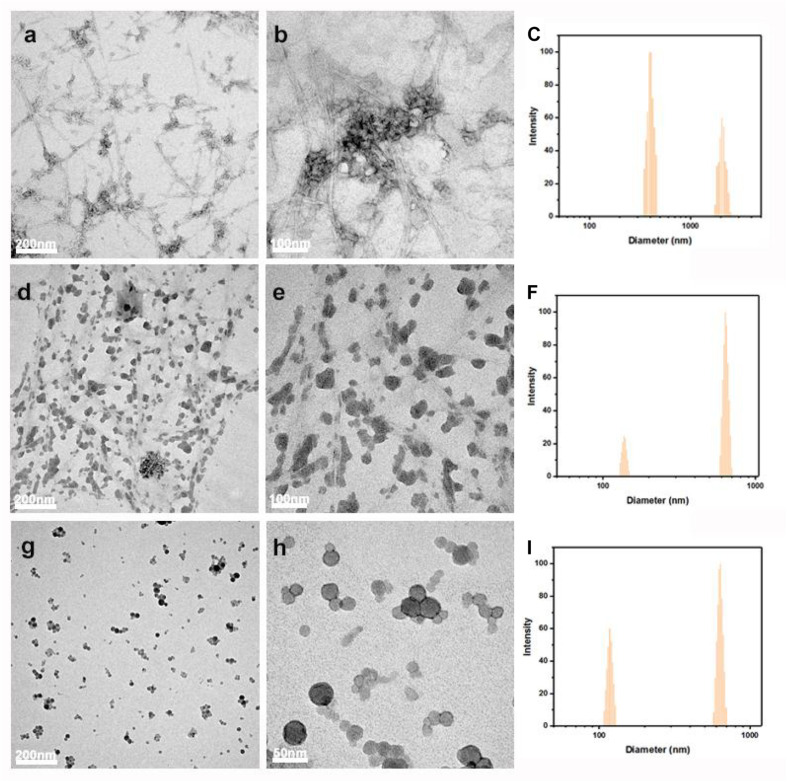
TEM images and the measured size distribution by DLS of LCNFs-48 h **(a–c)**; LCNFs-72 h **(d–f)**; and LNPs-108 h **(g–i)**.

As shown in Figure 3, the morphology of LCNFs and LNPs was further examined by TEM analysis and the particle size was evaluated by DLS. From the TEM images, we can see the coexistence of nanofibers and LNPs for the samples of LCNFs-48 h and LCNFs-72 h. It should be noted that the DLS can only be used as a reference for particle distribution, not as a basis for the real particle size. As shown in Figures 3a,b, a large number of nanofibers with a clear fiber boundary can be seen and some of the nanofibers are anchored with some LNPs. From Figures 3d,e, most of the nanofibers are covered with LNPs, and the fiber boundary can be hardly observed. It can be seen from Figures 3g,h that LNPs with a diameter less than 50 nm can be obtained from EHRs-108 h, but it is speculated that there is still a very small amount of cellulose nanofibers embedded among them according to the component analysis results of EHRs-108 h and Figure 3i.

In addition, Figure 3 indicates the measured particle size decreased with the increased lignin content in the samples. This phenomenon can be explained as follows: (1) the measured data of the sample containing nanocellulose would be larger than the actual, because of the large draw ratio. While, lignin nanoparticles can reduce the interference of draw ratio because of its near spherical shape, which lead to the measurement is closer to the actual. (2) As the lignin content increased in the samples, the possibility of attraction among the nanofibrils decreased, contributing to the separation of nanofibrils. Consequently, the measured data decreased with the increase of lignin content (Ferrer et al., 2012; Dong et al., 2020b; Pei et al., 2020; Zheng et al., 2021a).

### Thermal Stability of LCNFs and LNPs

**FIGURE 4:**
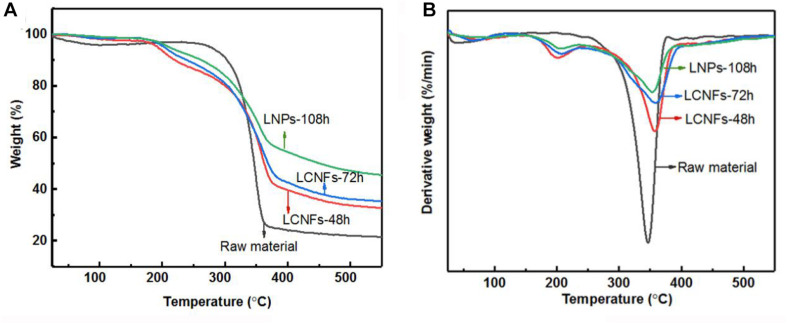
TG **(A)** and DTG **(B)** curves of LCNFs-48 h, LCNFs-72 h, LNPs-108 h, and raw material.

The thermal stability of the as-prepared LCNFs, LNPs, and raw materials was analyzed by TG analysis, and the results are shown in Figure 4. As shown in the TG and DTG curves, all samples mainly have two stages of thermal decomposition; one is before 250°C, and the other is between 250 and 400°C. For the raw material, only one weight loss peak was observed at the temperature of 350°C (T_max_), at which the weight loss rate was around 17.5%/min. In comparison, the DTG of the LCNFs and LNPs illustrated that these samples containing the high lignin content had two weight loss peaks, and one was around 209°C and the other was around 360°C. It can also be observed from these curves that the weight loss rate at T_max_ decreased gradually along with the increase of lignin content. According to the reported literatures, the lignin decomposed slower and in a broad range (between 160 and 900°C), while cellulose decomposition usually occurred in the range of 300–400°C. The reason for the decrease of the weight loss rate at T_max_ is also related to the content of cellulose in the samples. The content of cellulose in raw material was relatively high, resulting in the high degradation rate of cellulose. However, the content of cellulose in LCNFs and LNPs decreased with the prolonging of enzymatic hydrolysis time, causing the weight loss rate decreased at T_max_. In addition, the comparison of residual carbon produced by lignin was also consistent with the previous results (Wang et al., 2018).

### Chemical and Crystalline Structures of LCNFs and LNPs

**FIGURE 5:**
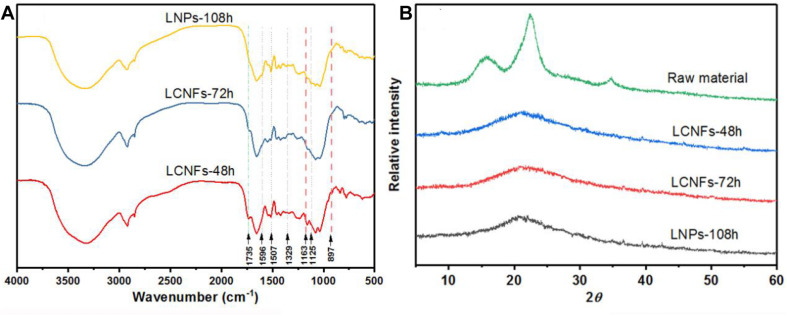
**(A)** FTIR spectra of the LCNFs-48 h, LCNFs-72 h, and LNPs-108 h. **(B)** X-ray diffraction patterns of raw material and the LCNFs and LNPs samples.

Figure 5A shows the FT-IR spectra of LCNF and LNP samples. Since no chemical reaction was performed on the lignin or cellulose during the high homogenization, all the spectra were very similar to the characteristic peak of lignin. The typical aromatic ring peaks of lignin fraction at 1,596 and 1,507 cm^–1^ were also evident (Schwanninger et al., 2004). In addition, peaks at 1,125 and 1,329 cm^–1^ were also observed, which were assigned to the condensed guaiacyl units and syringyl units of lignin, respectively (Rana et al., 2009). The peak at 1,735 cm^–1^ (C=O vibration) was observed in EHRs-48 h and EHRs-72 h, indicating the remaining of xylan in short-time EHRs (Sirvio et al., 2016). The characteristic peaks of cellulose were observed at 1,163 and 897 cm^–1^ in LCNFs-48 h and LCNFs-72 h, which was not obvious for LNPs-108 h (Schwanninger et al., 2004).

XRD patterns of the raw material and the LCNF and LNP samples are shown in Figure 5B. The raw material displays main characteristic diffraction peaks at 14.6°, 16.5°, 22.6°, and 34.6°, corresponding to the (1–10), (110), (200), and (004) lattice planes, respectively, indicating that the raw materials conform to the cellulose I structure (Sun et al., 2015; Du et al., 2020). On the contrary, the XRD patterns of the LCNFs and LNPs just show a broad amorphous peak, and the highest peak was around 20.5°. This could be ascribed to the removal of cellulose and the amorphous nature of lignin (Peng et al., 2018).

### Dispersion Stability

**FIGURE 6:**
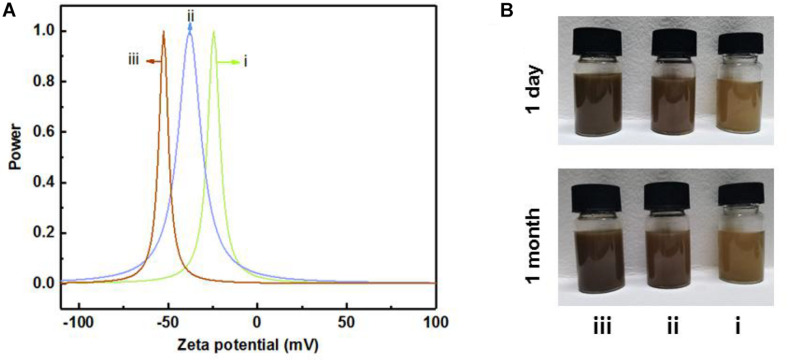
The zeta potential **(A)** and corresponding dispersibility in water **(B)** of LCNFs-48 h (i), LCNFs-72 h (ii), and LNPs-108 h (iii).

The dispersion stability of LCNF and LNP suspension was evaluated by the zeta potential. It is well documented that the closer the value measured by the zeta potential was to zero, the easier the dispersion was to agglomerate and settle, and the worse the relative stability is (Herrera et al., 2018). As shown in Figure 6A, the zeta-potential value of the dispersion formed by LNPs-108 h was around −52 mV, indicating the best stability. It has been demonstrated that the stability of the solution would increase with the increase of lignin content, for it would improve the fibrillation process (Lahtinen et al., 2014), which increases the surface charge of the material, resulting in repulsion between the fibrils thus allowing an easier separation from each other (Rojo et al., 2015). Moreover, as shown in Figure 6B, all the samples exhibit very stable colloidal suspensions in aqueous media for over 1 month, indicating excellent dispersibility.

## Proposed Process for the Full Utilization of Corncob

**FIGURE 7:**
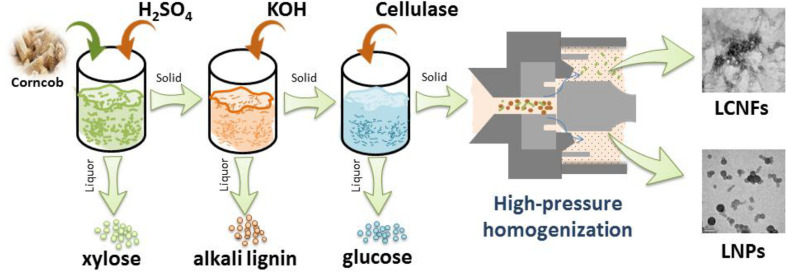
Proposed process for the full utilization of corncob.

Herein, based on the results obtained in this work and our previous work, we propose the full component utilization of corncob to provide a reference for the full utilization of other similar agricultural wastes. Generally, hemicellulose is extracted firstly from corncob in the industry, which mainly involves acid treatment. On the basis of the research that we had finished (Xie et al., 2018b; Chi et al., 2019), alkali treatment was a convenient way to effectively separate alkali lignin from agricultural lignocellulosic waste and promote the downstream enzymatic hydrolysis for the production of fermentable sugars. The current study demonstrated that the EHRs could be an attractive material for the preparation of LCNFs or LNPs, which might find applications in diverse fields such as reinforcing nanofillers and Pickering emulsions. In order to show the operation involved in the proposed process more intuitively, Figure 7 is proposed to facilitate understanding. As shown in the proposed flowchart, the solid residue produced in the previous part is used to produce the desired substance in the next step. Specifically, xylose (originated from hemicellulose) was obtained by acid treatment, alkali lignin was obtained by alkali treatment, monosaccharide (derived from cellulose) was obtained by enzymatic hydrolysis, and LCNFs with different lignin contents or LNPs could be obtained by homogenization. Thus, the comprehensive utilization of the whole components of corncob can be realized.

## Conclusion

In summary, high LCNFs and LNPs were successfully prepared from EHRs of corncob residues via high-pressure homogenization. During enzymatic hydrolysis process, with the increase of enzymolysis time, the lignin content increased and the cellulose content decreased resulting in the EHRs with controllable chemical compositions. It was found that the lignin content had a great influence on the morphology of the obtained nanoparticles after homogenization. The higher the lignin content in the EHRs, the smaller the size and the higher the absolute zeta potential of the obtained nanoparticles observed. This work not only promotes the development of lignocellulose-based nanomaterials but also provides a promising full utilization for the agricultural residue that simultaneously achieves the maximum use of the whole biomass.

## Data Availability Statement

The original contributions presented in the study are included in the article/supplementary material, further inquiries can be directed to the corresponding author/s.

## Author Contributions

RX, HD, HW, MZ, MW, CL, GY, and S-EC: investigation. CS, S-EC, and BL: supervision. RX and HD: writing—original draft. XZ, BL, S-EC, and CS: writing—review and editing. All authors contributed to the article and approved the submitted version.

## Conflict of Interest

The authors declare that the research was conducted in the absence of any commercial or financial relationships that could be construed as a potential conflict of interest.
